# Overexpression of N-myc downstream-regulated gene 1 inhibits human glioma proliferation and invasion via phosphoinositide 3-kinase/AKT pathways

**DOI:** 10.3892/mmr.2015.3492

**Published:** 2015-03-13

**Authors:** WEI MA, MENG NA, CHONGYANG TANG, HAIYANG WANG, ZHIGUO LIN

**Affiliations:** Department of Neurosurgery, The First Affiliated Hospital of Harbin Medical University, Harbin, Heilongjiang 150001, P.R. China

**Keywords:** glioma, N-myc downstream-regulated gene 1, apoptosis, metastasis, AKT

## Abstract

N-myc downstream-regulated gene 1 (NDRG1) was previously shown to exhibit low expression in glioma tissue as compared with that in normal brain tissue; however, the role of NDRG1 in human glioma cells has remained to be elucidated. The present study used the U87 MG and SHG-44 human glioma cell lines as well as the normal human astrocyte cell line 1800, which are known to have differential NDRG1 expression. Small interfering (si)RNA targeting NDRG1, and NDRG1 overexpression vectors were transfected into the SHG-44 and U87 MG glioma cells, respectively. Cell proliferation, invasion, apoptosis and cell cycle arrest were subsequently examined by MTT assay, transwell chamber assay, flow cytometry and western blot analysis, respectively. Furthermore, a subcutaneous tumor mouse model was used to investigate the effects of NDRG1 on the growth of glioma cells *in vivo*. Overexpression of NDRG1 was shown to inhibit cell proliferation and invasion, and induce apoptosis in the U87 MG glioma cells, whereas NDRG1 downregulation increased proliferation, suppressed apoptosis and promoted invasion of the SHG-44 glioma cells. In addition, in the subcutaneous tumor mouse model, overexpression of NDRG1 in U-87 MG cells suppressed tumorigenicity *in vivo*. The findings of the present study indicated that NDRG1 is required for the inhibition of gliomagenesis; therefore, targeting NDRG1 and its downstream targets may represent novel therapies for the treatment of glioma.

## Introduction

Malignant glioma is the most common type of primary brain tumor (1), which is responsible for ~1/3 of central nervous system intrinsic neoplasms in adults and children (2–4). Gliomas are aggressive tumors that possess a tendency to invade the surrounding brain tissue. In addition, glioma cells are proliferate rapidly and are often resistant to common forms of treatment, including surgical resection, chemotherapy and radiotherapy (5,6). However, the factors that govern the progression and invasion of glioma are currently not well understood.

N-myc downstream-regulated gene 1 (NDRG1) was initially identified as gene that was upregulated in N-myc knockout mouse embryos, and it was able to be repressed by N-myc and c-myc (7,8). Since its initial identification, NDRG1 has been isolated by numerous laboratories under various physiological conditions (9,10). NDRG1 is a 43-kD protein, which is comprised of 394 amino acids and is known to be highly conserved among multicellular organisms. NDRG1 is predominantly cytosolic and is ubiquitously expressed in tissues in response to cellular stress signals (11,12). There are currently known to be four members of the human NDRG family: NDRG1, NDRG2, NDRG3 and NDRG4. The amino acid homology among each member is ~57–65% (13,14).

mRNA and protein expression of NDRG1 was found to be decreased in primary cancer and metastatic cells, including colon (15,16), prostate (17,18), breast (18) and esophageal squamous cancer (19), as well as glioma (20), as compared with that in normal cells. In addition, NDRG1 expression has been shown to be upregulated in mouse skin carcinomas and in hyperplastic skin epithelium, as compared with that in normal mouse skin (13). Numerous studies have demonstrated that NDRG1 is associated with cellular growth (21–23), differentiation (13,24), tumorigenesis (25), metastasis and poor clinical outcome (26–28) in certain tumors.

Phosphoinositide 3-kinase (PI3K)/Akt signaling is an important survival/proliferative pathway involving various growth factors, cytokines, and activation of receptors (29). Akt is upregulated in numerous types of human cancer, including glioma, and links to oncogenesis to alter cellular functions (29). For example, Akt promotes tumor proliferation by inhibiting apoptosis (29); Akt is involved in cell cycle regulation by preventing degradation of cyclin D1 (30), and by negatively regulating p27 (31) and p21 (32). The present study aimed to determine whether NDRG1 could inhibit proliferation and invasion of glioma through the PI3K/Akt signaling pathway.

In a previous study, Sun *et al* (20) demonstrated that NDRG1 expression was downregulated in tissue specimens from high-grade gliomas, as compared with that in tissue from low-grade gliomas and normal brain tissue. These results suggested that NDRG1 may be an intrinsic regulator of gliomagenesis. In addition, NDRG1 was shown to negatively regulate myc protein (7). However, the role of NDRG1 in human glioma has yet to be fully elucidated. The present study aimed to determine the expression and pathological roles of NDRG1 in human glioma, and to investigate whether NDRG1 could serve as a potential target for the treatment of glioma.

## Materials and methods

### Cell culture

The U87 MG and SHG-44 human malignant glioma cell lines, and the normal human astrocyte cell line 1800 were obtained from the Cell Library of the Chinese Academy of Sciences (Shanghai, China). The U87 MG and SHG-44 cells were cultured in Dulbecco’s modified Eagle’s medium (DMEM; Gibco-BRL, Invitrogen Life Technologies, Carlsbad, CA, USA) containing 10% fetal bovine serum (FBS), 2 mM L-glutamine, and 100 U/ml penicillin-streptomycin (all Gibco-BRL) at 37°C in an atmosphere containing 5% CO_2_. The normal astroctyes (1800) were cultured in modified RPMI-1640 (HyClone Laboratories, Inc., Logan, UT, USA) supplemented with 10% FBS, 2 mM L-glutamine, and 100 U/ml penicillin-streptomycin, at 37°C in an atmosphere containing 5% CO_2_. The medium was replaced every 3–4 days, and the cultures were split using 0.25% trypsin (Gibco-BRL).

### Transfections

Small interfering (si) RNA targeting human NDRG1 (siNDRG1) (sense 5′-GCUGAAGCUCGUCAGUU CACCAUCC-3′ and anti-sense 5′-GGAUGGUGAACUGACGAGCUUCAGCAC-3′), and negative control si RNA (si NC) (sense 5′-UUCUCCGAACGUGUCACGU-3′ and antisense 5′-ACGUGACACGUUCGGAGAA-3′), were purchased from Biomics Biotechnologies Co., Ltd. (Nantong, China). The siRNA were transfected into SHG-44 cells using Lipofectamine^®^ reagent (Invitrogen Life Technologies, Carlsbad, CA, USA), according to the manufacturer’s instructions. Human pLPCX-NDRG1 and pLPCX were purchased from Biowot Technologies (Shenzhen, China). To generate a retrovirus, the packaging line gp2–293 (Cell Library of the Chinese Academy of Sciences, Shanghai, China) was co-transfected with pCMV-VSVG (Adgene, Cambridge, MA, USA), and either pLPCX or pLPCX-NDRG1, using FuGENE^®^ 6 transfection reagent (Roche Diagnostics Corp., Indianapolis, IN, USA). Retrovirus-containing conditioned medium was harvested, filtered through a 0.45-*μ*m syringe filter unit (EMD Millipore, Billerica, MA, USA), and used to trans-duce U87 MG cells, according to standard procedures (33). Following retroviral infection, single-cell clonal isolates were selected in the presence of puromycin (Sigma-Aldrich, St. Louis, MO, USA).

### MTT assay

Normal untransfected cells and transfected cells (48 h post-transfection) were seeded in 96-well plates at a density of 2×10^3^ cells/well. After 24, 48 and 72 h, the medium was replaced with 200 *μ*l DMEM supplemented with 10% FBS and 0.5 mg/ml MTT (Sigma-Aldrich), and the cells were incubated at 37°C in a 5% CO_2_ incubator for 4 h. Subsequently, the medium was removed, and the reduced MTT was solubilized in 100 *μ*l/well dimethyl sulfoxide (Sigma-Aldrich). The absorbance was measured at a wavelength of 570 nm using an iMark microplate reader (Bio-Rad Laboratories, Inc., Hercules, CA, USA).

### Cell cycle and apoptosis analysis

Cell cycle and apop-tosis assays were performed as described previously (34). Annexin V-fluorescein isothiocyanate apoptosis kit and cell cycle analysis kit (both from BD Biosciences, Franklin Lakes, NJ, USA) were used according to the manufacturer’s instructions. The cells were analyzed on a FACSCalibur flow cytometer (BD Biosciences).

### Cell invasion assay

Invasion was measured using 24-well BioCoat cell culture inserts (BD Biosciences) with an 8-*μ*m-porosity polyethylene terephthalate membrane coated with Matrigel Basement Membrane Matrix (BD Biosciences). The invasion assay was performed as previously described (35).

### Immunofluorescence

Briefly, 2×10^5^ cells were seeded onto coverslips, fixed with 4% (w/v) paraformaldehyde (Sigma-Aldrich) for 10 min and permeabilized with 0.1% (v/v) Triton X-100 (Sigma-Aldrich) for 5 min at room temperature. The cells were subsequently incubated at 4°C overnight with the following primary antibodies: Rabbit polyclonal anti-human N-cadherin (1:100; cat. no. ab18203; Abcam, Cambridge, MA, USA) and mouse monoclonal anti-human vimentin (1:100; cat. no. ab8978; Abcam). The cells were then incubated with polyclonal Alexa Fluor^®^ 555-conjugated goat anti-mouse (1:200; cat. no. A21422; Invitrogen Life Technologies) and polyclonal Alexa Fluor^®^ 488-conjugated goat anti-rabbit (1:200; cat. no. A11008; Invitrogen Life Technologies) immunoglobulin G (IgG) secondary antibodies for 1 h at room temperature. The coverslips were then washed with phosphate-buffered saline (PBS; Sigma-Aldrich), and were mounted using an anti-fade mounting solution containing DAPI (Vector Laboratories, Inc., Burlingame, CA, USA). The images were visualized and captured using a fluorescent microscope (Eclipse Ni-E; Nikon Corporation, Tokyo, Japan).

### Western blot analysis

Western blot analysis was performed as described previously (34). Whole cell protein lysates were extracted using lysis buffer (Invitrogen Life Technologies) supplemented with a proteinase inhibitor mixture (Sigma-Aldrich) and PhosSTOP (Roche Diagnostics Corp.). Protein concentrations were determined using a bicinchoninic acid protein assay (Beyotime Institute of Biotechnology, Jiangsu, China). Protein lysates were mixed with 6X protein sample buffer [0.35 mol/l Tris-HCl (pH 6.8; Sigma-Aldrich), 30% glycerol (Sigma-Aldrich), 21.4% β-mercaptoethanol (Sigma-Aldrich), 10% SDS] and boiled for 5 min. A total of 25 *μ*g protein was then loaded onto 10 or 12% SDS-polyacrylamide gels for electrophoresis, and transferred onto polyvinylidene difluoride membranes (EMD Millpore), at a constant voltage of 100 V. The membranes were then blocked with 10% nonfat milk (Wuhan Boster Biological Technology Ltd., Wuhan, China) at room temperature for 1 h and washed with a large volume of trisbuffered saline containing Tween [20 mmol/l Tris-HCl, 137 mmol/l NaCl (Sigma-Aldrich), 0.1% Tween 20 (Sigma-Aldrich)].

Monoclonal rabbit anti-human NDRG1 (dilution 1:1,000; cat. no. ab124689), monoclonal mouse anti-human cyclin D1 (dilution 1:1,000; cat. no. ab101430), polyclonal rabbit anti-human cyclin E (dilution 1:1,000; cat. no. ab7959), monoclonal mouse anti-human PCNA (dilution 1:2,000; cat. no. ab29) and monoclonal mouse anti-human Ki-67 (dilution 1:1,000; cat. no. ab6526) primary antibodies were purchased from Abcam. Monoclonal mouse anti-human AKT (dilution 1:2,000; cat. no. 2920), monoclonal rabbit anti-human p-AKT (Ser473) (dilution 1:1,000; cat. no. 4060), monoclonal rabbit anti-human Bcl-xL (dilution 1:1,000; cat. no. 2764), polyclonal rabbit anti-human Bcl-2 (dilution 1:1,000; cat. no. 2876), polyclonal rabbit anti-human Bax (dilution 1:1,000; cat. no. 2774), monoclonal rabbit anti-human cleaved-PARP (dilution 1:1,000; cat. no. 5625) and monoclonal rabbit anti-human cleaved-caspase-3 (dilution 1:1,000; cat. no. 9664) antibodies were purchased from Cell Signaling Technology, Inc. (Danvers, MA, USA). Polyclonal rabbit anti-human N-cadherin (dilution 1:1,000, cat. no. sc-7939), polyclonal rabbit anti-human E-cadherin (dilution 1:1,000; cat. no. sc-7870), monoclonal mouse anti-human vimentin (dilution 1:1,000; cat. no. sc-6260) and monoclonal mouse anti-human β-actin (dilution 1:3,000; cat. no. sc-47778) were purchased from Santa Cruz Biotechnology, Inc. (Dallas, Texas, USA). The membranes were incubated with the antibodies targeting NDRD1, cyclin D1, cyclin E, p-AKT (Ser473), Bcl-xL, Bcl-2, cleaved-PARP, cleaved-caspase-3, N-cadherin, E-cadherin and vimentin overnight at 4°C, and with the antibodies targeting PCNA, Ki-67, AKT, Bax and β-actin for 1 h at room temperature. Subsequently, the membranes were incubated with the secondary antibodies for 1 h at room temperature. The following secondary antibodies were used: Goat anti-mouse IgG-horseradish peroxidase (HRP) (dilution 1:2,000; cat. no. sc-2005) and goat anti-rabbit IgG-HRP (dilution 1:2,000; cat. no. sc-2004) from Santa Cruz Biotechnology, Inc. The blots were then assessed using a Pierce ECL western blotting substrate (Thermo Scientific, Rockford, IL, USA). The resulting bands were evaluated by densitometric measurement using ImageJ 1.46r (National Institutes of Health, Bethesda, MD, USA).

### Nude mouse xenograft studies

The study was approved by the Ethics Committee of The First Affiliated Hospital of Harbin Medical University (Harbin, China). A total of 14 female athymic nude mice (BALBc nu/nu; average weight 20 g; 6 weeks-old; Experimental Animal Laboratories, Shanghai, China) were used in all experiments (n=7/group). The mice were maintained in a specific pathogen-free environment and had *ad libitum* access to autoclaved food and water. The mice were maintained in a room at 20–22°C under a 12-hour light/dark cycle. Each mouse was injected subcutaneously with stably transfected U87 MG cells and control cells (1×10^6^). Tumor size was measured using a vernier caliper, and tumor volume (mm^3^) was calculated using the following standard formula: Tumor volume = length × width × height × 0.5236. All mice were sacrificed by CO_2_ inhalation six weeks after implantation, the tumor tissues were frozen immediately in liquid nitrogen and paraffin-embedded tumor tissue blocks were obtained for further analysis.

### Immunohistochemistry

Immunohistochemistry was performed using mouse monoclonal anti-Ki-67 (dilution 1:200; cat. no. ab6526; Abcam), rabbit monoclonal anti-cleaved-caspase-3 (dilution 1:200; cat. no. 9664; Cell Signaling Technology, Inc.) and rabbit polyclonal anti-CD31 (dilution 1:100; cat. no. ab28364; Abcam) antibodies. Briefly, tissue sections were deparaffinized in xylene (Sigma-Aldrich) and rehydrated with ethanol (Sigma-Aldrich). The tissue sections were subsequently incubated with 10% normal goat serum (Vector Laboratories, Inc., Burlingame, CA, USA in PBS (pH 7.5), followed by an overnight incubation at 4°C with the primary antibodies. The tissue sections were then stained with biotinylated secondary antibody (Vector Laboratories, Inc.) for 1 h at room temperature, followed by an incubation with Vectastain Elite avidin-biotin complex reagent (Vector Laboratories, Inc.) for 30 min. The peroxidase reaction was developed using diaminobenzidine (DAB kit; Vector Laboratories, Inc.) and the slides were counterstained with hematoxylin (Sigma-Aldrich).

### Statistical analysis

Statistical analysis was performed using GraphPad Prism version 4.02 software (GraphPad Software Inc., La Jolla, CA, USA) or SPSS 16.0 software (SPSS, Inc., Chicago, IL, USA). Values are expressed as the mean ± standard deviation. Comparisons between multiple groups were made using a one-way analysis of variance, followed by Dunnet’s t-test. P<0.05 was considered to indicate a statistically significant difference between values.

## Results

### NDRG1 is lowly expressed in glioma cells

**Figure 1:**
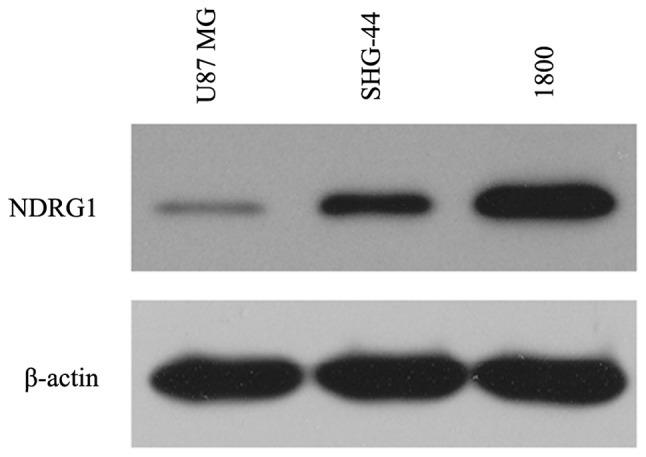
Protein expression levels of NDRG1 were measured in all cell lines by western blotting. The blot is representative of three independent experiments. β-actin was used as the internal control. NDRG1, N-myc downstream-regulated gene 1.

The present study examined the expression levels of NDRG1 in the established human glioma cell lines U87 MG and SHG-44, and in the normal astroglial cell line 1800. NDRG1 protein expression levels were low in the U87 MG and SHG-44 glioma cells, whereas high expression levels of NDRG1 were observed in the normal astroglial cell line (1800), as determined by western blotting. Furthermore, NDRG1 expression was lower in the U87 MG cells as compared with that in the SHG-44 cells (Fig. 1).

### NDRG1 inhibits cell proliferation in glioma cells

**Figure 2:**
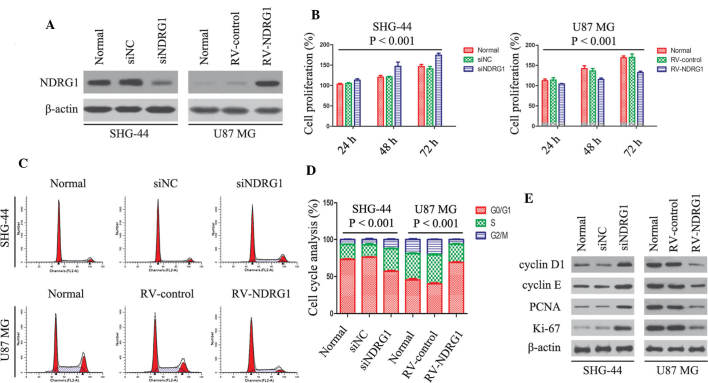
Effects of NDRG1 on cell proliferation, cell cycle-associated protein expression and cell cycle progression in SHG-44 and U87 MG glioma cells. (A) Western blot analysis showed that protein expression levels of NDRG1 were affected by transfection with siNDRG1 and RV-NDRG1. β-actin was used as the internal control. (B) Effects of NDRG1 knockdown and overexpression on the proliferation of glioma cells. Cell proliferation of untransfected cells and cells transfected with siNDRG1, siNC, RV-NDRG1 or RV-control was assessed by MTT assay. Values are expressed as the mean ± standard deviation of three independent experiments. (C and D) Cell cycle analysis of glioma cells transfected with siNDRG1 or RV-NDRG1 demonstrated an increase in the number of siNDRG1-transfected SHG-44 cells in S phase, and an increase in the number of RV-NDRG1-transfected U87 MG cells in G_0_/G_1_ phase. The percentage of cells in each phase of the cell cycle is presented as the mean ± standard deviation from three independent experiments. (E) Changes in protein expression levels of proliferation indices: Ki-67, PCNA, cyclin D1 and cyclin E following transfection with siNDRG1 or RV-NDRG1. β-actin was used as the internal control. NDRG1, N-myc downstream-regulated gene 1; si, small interfering RNA; RV, retrovirus; NC, negative control; PCNA, proliferating cell nuclear antigen.

To determine whether NDRG1 expression had an effect on glioma cell progression, the present study used siNDRG1 to specifically knockdown NDRG1 expression, and used retroviral constructs expressing NDRG1 (RV-NDRG1) to enforce NDRG1 overexpression. NDRG1 expression levels were lowest in the U87 MG cells and were highest in the SHG-44 cells (Fig. 1). siNDRG1 was transfected into the SHG-44 cells in order to downregulate NDRG1 expression, and RV-NDRG1 was transfected into the U87 MG cells to enhance NDRG1 expression. Transfection with siNDRG1 knocked down NDRG1 expression levels in SHG-44 cells by ~80% within two days of transfection, whereas no decrease in NDRG1 expression was observed in the cells transfected with the siNC, as determined by western blot analysis (Fig. 2A). Furthermore, U87 MG cells transfected with RV-NDRG1 exhibited increased protein expression levels of NDRG1, as determined by western blotting (Fig. 2A).

To determine whether there was an association between NDRG1 expression and glioma cell proliferation, an MTT assay was performed. SHG-44 cells transfected with siNDRG1 exhibited a marked increase in cell proliferation as compared with that in the siNC and normal groups (P<0.001; Fig. 2B). In addition, there was a significant decrease in cell proliferation in the U87 MG cells transfected with RV-NDRG1 (P<0.001; Fig. 2B).

Flow cytometry revealed an increase in the number of siNDRG1-transfected SHG-44 cells in S phase at 72 h, as compared with that in the siNC group (P<0.001; Fig. 2C and D). Conversely, there was an increase in the number of RV-NDRG1-transfected U87 MG cells in G_0_/G_1_ phase (P<0.001; Fig. 2C and D). Concurrently, the expression levels of cell cycle arrest-associated proteins were examined by western blot analysis (Fig. 2E). The protein expression levels of cyclin D1, cyclin E, Ki-67 and PCNA were higher in the siNDRG1-transfected SGH-44 cells, as compared with those in the siNC-transfected and untransfected cells. Conversely, the expression levels were lower in the RV-NDRG1-transfected U87 MG cells as compared with those in untransfected cells, which was concordant with the results from the MTT and cell cycle assays. These results suggested that NDRG1 may inhibit cell proliferation and induce G_0_/G_1_ cell cycle arrest in glioma cells.

### NDRG1 increases the percentage of apoptotic glioma cells

**Figure 3:**
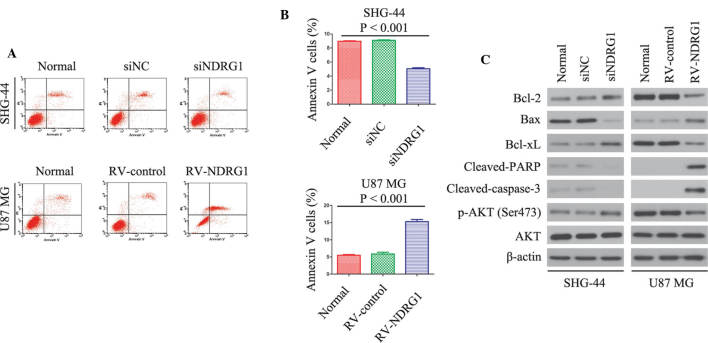
NDRG1 induces cell death in a caspase-dependent manner. NDRG1 affects constitutive and inducible p-AKT expression in SHG-44 and U87 MG glioma cells, and overexpression of NDRG1 downregulates the expression of anti-apoptotic proteins. (A and B) Flow cytometry results of annexin V-PI staining of glioma cells following transfection with siNDRG1 or RV-NDRG1. An increase in the percentage of apoptotic cells following transfection with RV-NDRG1 is shown. Values are expressed as the mean ± standard deviation of three independent experiments. (C) Changes in protein expression levels of anti- or pro-apoptotic proteins following transfection with siNDRG1 or RV-NDRG1. β-actin was used as the internal control. NDRG1, N-myc downstream-regulated gene 1; p, phosphorylated; si, small interfering RNA; RV, retrovirus; NC, negative control; Bcl-2, B-cell lymphoma 2; Bax, Bcl-2-associated X protein; xL, extra large; PARP; poly(ADP ribose) polymerase; PI, propidium iodide.

The number of apoptotic glioma cells was measured using an Annexin V/PI Apoptosis Detection kit and flow cytometry at three days post-transfection. RV-NDRG1-transfected U87 MG cells exhibited a relatively high rate of cell apoptosis as compared with that in the cells transfected with the scrambled control or the untransfected cells (P<0.001; Fig. 3A and B). Conversely, SHG-44 cells transfected with siNDRG1 exhibited a relatively low rate of apoptosis as compared with that of the untransfected cells (P<0.001; Fig. 3A and B).

In addition, western blot analysis was performed to determine the expression levels of the following apoptosis-associated proteins in glioma cells: Bcl-2, Bax, Bcl-xL, cleaved-PARP, cleaved-caspase-3, AKT and p-AKT. At three days post-transfection, the expression levels of Bcl-2 and Bcl-xL were significantly decreased in the RV-NDRG1-transfected U87 MG cells as compared with the cells transfected with the RV-control, whereas the expression levels of Bax, cleaved-PARP and cleaved-caspase-3 were increased. In addition, RV-NDRG1-transfected U87 MG cells exhibited lower expression levels of p-AKT, whereas the expression levels of total AKT were unaffected (Fig. 3C). In accordance with the data from the cells overexpressing NDRG1, three days post-transfection, Bcl-2, Bcl-xL and p-AKT expression were upregulated, and the expression levels of Bax, cleaved-PARP and cleaved-caspase-3 were decreased in the siNDRG1-trans-fected SHG-44 cells, but not in the control group (Fig. 3C). These results indicated that the NDRG1-dependent increase in the rate of glioma cell apoptosis may be partially mediated by regulation of Bcl-2, Bcl-xL, Bax, cleaved-PARP, cleaved-caspase-3 and p-AKT.

### NDRG1 inhibits glioma cell invasion in vitro

**Figure 4:**
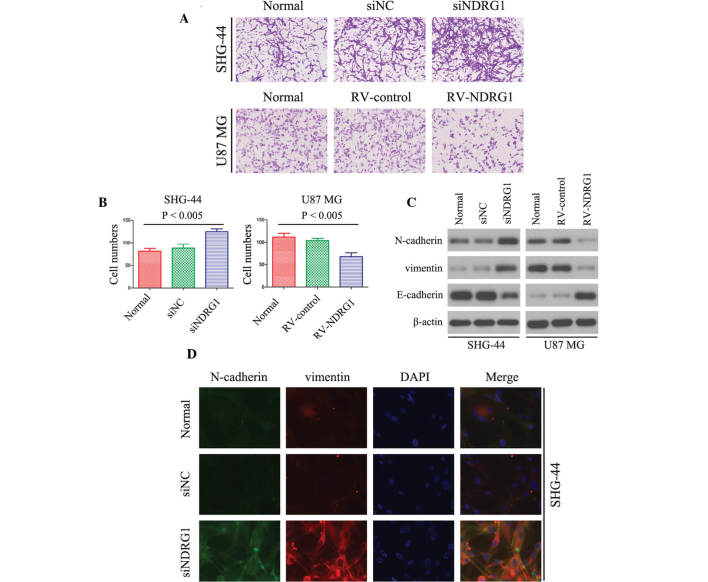
NDRG1 affects the invasive potential of SHG-44 and U87 M glioma cells *in vitro*. (A and B) Cell invasion experiments demonstrated that transfection with RV-NDRG1 significantly inhibited the invasive capacity of U87 MG cells, and transfection with siNDRG1 promoted the invasive capacity of SHG-44 cells (stained with hematoxylin). (A) Magnification, ×40; (B) values are expressed as the mean ± standard deviation of experiments performed in triplicate. (C) Protein expression levels of N-cadherin, vimentin and E-cadherin in RV-NDRG1-transfected U87 MG cells and siNDRG1-transfected SHG-44 cells. β-actin was used as the internal control. (D) Single and merged images were taken to show immunofluorescence staining of N-cadherin (green) and vimentin (red), accompanied by nuclear staining (blue) with DAPI. NDRG1, N-myc downstream-regulated gene 1; si, small interfering RNA; RV, retrovirus; NC, negative control.

The association between NDRG1 expression and glioma cell invasion was detected using a Matrigel invasion assay. The number of invaded RV-NDRG1-transfected U87 MG cells was significantly lower as compared with that in the cells in the RV-control and untransfected groups (P<0.005; Fig. 4A and B), thus suggesting that the percentage of invaded cells decreased in cells overexpressing NDRG1. Furthermore, the invasiveness of siNDRG1-transfected SHG-44 cells was increased as compared with that of untransfected cells (P<0.005; Fig. 4A and B).

Vimentin, N-cadherin and E-cadherin have essential roles in the invasion of tumor cells (36). Therefore, the present study examined the expression levels of vimentin, N-cadherin and E-cadherin in glioma cells by western blotting. The protein expression levels of vimentin and N-cadherin were downregulated in RV-NDRG1-transfected U87 MG cells as compared with those in the control groups, and E-cadherin expression levels were upregulated (Fig. 4C). In addition, in siNDRG1-transfected SHG-44 cells, the expression levels of vimentin and N-cadherin were significantly increased, and expression levels of E-cadherin were significantly decreased (Fig. 4C). As shown by immunofluorescence (Fig. 4D), transfection with siNDRG1 markedly increased N-cadherin and vimentin expression in SHG-44 cells, which was in concordance with the results of the western blot analysis. These results suggested that NDRG1 may inhibit glioma cell invasion *in vitro*.

### NDRG1 overexpression inhibits glioma tumor growth in vivo

**Figure 5:**
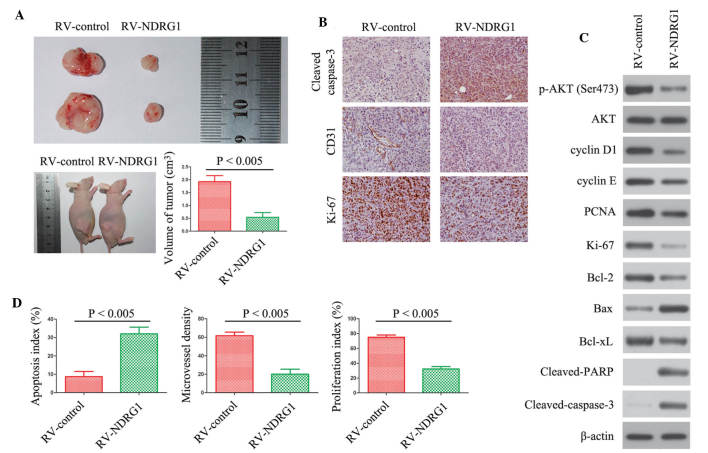
NDRG1 overexpression inhibits proliferation of glioma *in vivo*. (A) Photomicrographs were taken of U87 MG xenograft tumors grown in nude mice. Representative images of one mouse from each group are presented. Tumor volumes in the mice with tumors overexpressing NDRG1 were smaller, as compared with those in the control mice. Values are expressed as the mean ± standard deviation of experiments performed in triplicate. (B) Tumors from the different groups were immunostained for cleaved caspase-3, CD31 and Ki-67. Images are representative of three independent experiments. (C) Western blotting was performed to detect the protein expression levels of the indicated molecules from tumor samples. β-actin was used as the internal control. (D) Quantification of immunostaining in B. CD31-stained microvessels were counted to record microvessel density, apoptotic cells were counted to give the apoptosis index and cells expressing Ki-67 were counted to calculate the proliferation index. NDRG1, N-myc downstream-regulated gene 1; RV, retrovirus; Bcl-2, B-cell lymphoma 2; Bax, Bcl-2-associated X protein; xL, extra large; PARP; poly ADP ribose polymerase; PCNA, proliferating cell nuclear antigen; p, phosphorylated; RV, retrovirus.

The present study further examined the effects of NDRG1 on glioma growth by establishing a U87 MG xenograft nude mouse glioma model. Mice injected with glioma cells over-expressing NDRG1 exhibited significantly smaller tumors as compared with those in the control group (P<0.005; Fig. 5A). Immunohistochemical analysis was used to stain Ki-67 to determine cell proliferation; cleaved-caspase-3 to detect apoptotic cells; and CD31 to detect tumor microvessels. There were fewer Ki-67 positive cells, more apoptotic cells and fewer CD31-stained vessels in tumors overexpressing NDRG1, as compared with the control tumors (Fig. 5B). In addition, the expression levels of the same proteins were assessed *in vitro* by western blotting (Fig. 5C). The protein expression levels of p-AKT (ser473), cyclin D1, cyclin E, PCNA, Ki-67, Bcl-2 and Bcl-xL were decreased in the NDRG1 overexpressing cells, as compared with the control cells (Fig. 5C). In addition, the expression levels of Bax, cleaved-PARP and cleaved-caspase-3 were increased in the cells overexpressing NDRG1. Fig. 5D shows the quantification of the immunohistochemical analyses (P<0.005). The quantification revealed a 2-fold decrease in the number of Ki-67 positive cells in the RV-NDRG1 group, a >3-fold increase in the number of apoptotic cells in the RV-NDRG1 group and a 3-fold decrease in microvessel formation in the RV-NDRG1 group (P<0.005). These results indicate the functional significance of NDRG1, and its high propensity to inhibit proliferation in glioma.

## Discussion

NDRG1 has been associated with numerous cellular processes, including cell cycle, apoptosis and differentiation (12,24,37). Differentiation of various cancer cell lines *in vitro* has been shown to induce the expression of NDRG1 (38–40). In addition, the expression of NDRG1 in tumors has been shown to be associated with an improved outcome (41). Previous studies have suggested an important role for NDRG1 expression in numerous types of cancer (13,15,16,18). In addition, NDRG1 has often been shown to be downregulated in numerous types of human malignancy, including prostate (18), breast (26) and colon cancer (15). These observations suggested that NDRG1 may potentially act as a tumor suppressor in certain types of cancer.

The roles of NDRG1 are beginning to be elucidated in multiple malignancies; however, its function in brain tumori-genesis remained to be fully elucidated. Therefore, the present study aimed to investigate whether the expression of NDRG1 was associated with the progression of malignant glioma. A previous study demonstrated that the downregulation of NDRG1 is significantly associated with a higher World Health Organization tumor grade, and a worse overall survival rate (20). However, the mechanisms underlying the effects of NDRG1 on glioma tumorigenesis had yet to be elucidated. Therefore, it is required to study the role of NDRG1 in the regulation of glioma cell growth, survival and invasion.

In order to elucidate the role of NDRG1 in glioma, RV-NDRG1 was transiently transfected into U87 MG glioma cells. In the present study MTT, flow cytometric and invasive assays demonstrated that upregulation of NDRG1, following transfection of cells with RV-NDRG1, resulted in the inhibition of cell proliferation, induction of apoptosis and suppression of invasiveness. These results suggested that NDRG1 has an important role in the regulation of gliomagenesis. Further evidence regarding this finding was obtained from glioma cells with NDRG1 knockdown. Transfection of SHG-44 cells with siNDRG1 was used to silence NDRG1 expression, which resulted in increased cell proliferation and invasiveness, as well as decreased levels of apoptosis. Furthermore, in a subcutaneous mouse tumor model, NDRG1 overexpression significantly reduced subcutaneous tumor growth.

To provide further evidence regarding the mechanisms underlying the effects of NDRG1 on glioma cells, the present study examined the expression levels of proteins associated with glioma cell proliferation (cyclin D1, cyclin E, Ki-67 and PCNA), apoptosis (Bcl-2/Bax, Bcl-xL, cleaved-PARP, cleaved-caspase-3, p-AKT and AKT), and invasion (N-cadherin, vimentin and E-cadherin) by western blot analysis. The protein expression levels of Ki-67, a biological tumor marker that indicates changes in tumor proliferation (42), were reduced in the RV-NDRG1-transfected U87 MG cells, and increased in the siNDRG1-transfected SHG-44 cells. In addition, changes in the expression levels of PCNA, another well-known proliferation marker (43), were similar to those of Ki-67 in the RV-NDRG1-transfected U87 MG and the siNDRG1-transfected SHG-44 cells.

The protein expression levels of Bcl-2 have previously been shown to be associated with apoptosis in numerous cell types, including glioma cells (44). The results of the present study demonstrated that the downregulation of NDRG1 induced an increase in the protein expression levels of Bcl-2, and also significantly decreased the expression levels of Bax. The PI3K/Akt pathway is important in gliomas (45). Numerous studies have been performed to demonstrate that the p-Akt expression levels were elevated in gliomas *in vitro* and *in vivo*, and this expression was revealed to be correlated with the loss of phosphatase and tensin homolog (46,47). In glioma tumor samples, elevated p-Akt has been demonstrated to be associated with a worse prognosis (48). The present study revealed that NDRG1 knockdown also induced AKT phosphorylation, whereas it did not affect the expression of total AKT. Therefore, the increase in p-AKT, together with the increased expression of Bcl-2, may explain why cells with low NDRG1 expression are resistant to chemotherapy-induced apoptosis (49).

Another key characteristic of glioma cells, besides rapid proliferation and resistance to apoptosis, is their invasion into the surrounding healthy brain tissue (50). It has previously been suggested that vimentin, N-cadherin and the invasive marker E-cadherin are present at markedly elevated levels in numerous glioma cell lines and surgically resected specimens (51). The present study demonstrated a negative correlation between vimentin, N-cadherin and NDRG1 expression, and a positive correlation between E-cadherin and NDRG1 expression. These results indicated that the overexpression of NDRG1 may inhibit the invasiveness of glioma cells through modulation of vimentin, N-cadherin and E-cadherin. Poor histological differentiation is a property of malignancy.

The results of the nude mouse xenograft studies also confirmed that overexpression of NDRG1 was able to significantly inhibit proliferation and microvessel formation *in vivo*. Immunohistochemical analysis demonstrated that the expression of Ki-67 and CD31 were decreased in the tumor tissue, whereas the expression of cleaved-caspase-3 was increased in the RV-NDRG1 group. Western blotting also demonstrated that overexpression of NDRG1 resulted in increased expression levels of Bax, cleaved-PARP, cleaved-caspase-3 and decreased the expression levels of p-AKT (ser 473), cyclin D1, cyclin E, PCNA, Ki-67, Bcl-2 and Bcl-xL. The present study demonstrated that NDRG1 is important in gliomas. However, it remains to be elucidated why NDRG1 has a low level of expression in glioma. Specific signaling pathways may inhibit the expression of NDRG1, or NDRG1 may be degraded by certain microRNAs. Therefore, further studies are required to elucidate these questions.

In conclusion, the present study demonstrated that NDRG1 is lowly expressed in glioma cells. In addition, the results demonstrated that NDRG1 may have an important role in the regulation of growth and invasion of glioma cells. These findings indicated that NDRG1 may serve as a potential diagnostic or prognostic marker, and a novel therapeutic target in the treatment of glioma.
